# Dedifferentiated adamantinoma of long bones: a case report and literature review

**DOI:** 10.3389/fonc.2025.1559965

**Published:** 2025-08-11

**Authors:** Xiaofei Xiu, Jiajia Li, Lisha Duan, Xiaomei Wang, Zinan Guo, Xuelan Xiao, Qianhui Han, Feng Gao

**Affiliations:** ^1^ Department of Pathology, the Hebei Medical University Third Hospital, Hebei, Shijiazhuang, China; ^2^ Department of Economics and Management, the Hebei Vocational College of Labour Relations, Hebei, Shijiazhuang, China; ^3^ Department of Radiology, the Hebei Medical University Third Hospital, Hebei, Shijiazhuang, China

**Keywords:** adamantinoma, dedifferentiated, primary bone tumor, chondrosarcoma, bone neoplasm

## Abstract

We present a rare case of dedifferentiated adamantinoma. The 48-year-old male patient was initially diagnosed with classic adamantinoma in the right fibula and underwent curettage. Two years postoperatively, the patient presented with progressive swelling and pain in the same region. Imaging disclosed an expansile lesion with mixed lytic and sclerotic changes involving the medulla and adjacent soft tissue. A biopsy was performed, and a diagnosis of classic adamantinoma was made. Considering the significant clinical symptoms and imaging features, a below-knee amputation was performed. Histologically, the tumor exhibited two distinct patterns: classic adamantinoma juxtaposed to a dedifferentiated component, characterized by chondrosarcomatous areas with moderate cellularity and abundant hyaline cartilage matrix. Myxoid areas containing malignant cartilage cells of myxoid chondrosarcoma were also observed. Immunohistochemically, the dedifferentiated areas showed complete loss of epithelial markers. At a two-year follow-up, the patient remains free of recurrence or metastasis. Dedifferentiated adamantinoma is an exceedingly rare primary bone tumor malignancy lacking specific clinical manifestations. In our case, the initial biopsy specimen revealed only epithelial cells, leading to an underdiagnosis of classic adamantinoma until further sampling revealed the dedifferentiated component. It underscores the importance of thorough sampling, close radiologic-pathologic correlation, and a multidisciplinary framework for accurate diagnosis and optimal management of complex bone tumors. Early recognition of the dedifferentiated features can guide appropriate surgical management and improve patient outcomes.

## 1 Introduction

Adamantinoma of long bones is a rare, locally aggressive, or malignant primary bone neoplasm characterized by variable epithelial cells embedded within an osteofibrous stroma. Representing fewer than 1% of primary bone tumors, it exhibits a slight male predominance (male-to-female ratio ~ 5:4), although younger patients are more often female (1). Most cases arise between the second and fifth decades of life, commonly involving the mid-diaphysis of the tibia (2). Due to its morphological variability, the precise etiology remains unclear. Proposed etiologies include embryonal displacement or traumatic implantation of epithelial cells, though a leading hypothesis posits mesenchymal-epithelial transformation arising from the fibrous stroma (3, 4). Adamantinoma comprises three subtypes: classic, osteofibrous dysplasia-like (OFD-like), and dedifferentiated adamantinoma. Dedifferentiated adamantinoma is the rarest subtype, defined by concurrent classic adamantinoma and high-grade sarcomatous dedifferentiation, such as pleomorphic/spindle cell sarcoma and osteosarcoma. Up to now, 12 cases of dedifferentiated adamantinoma have been reported. Owing to the limited number of reported cases, its precise histogenesis and pathogenesis remain elusive.

Herein, we report a rare case of dedifferentiated adamantinoma with an unusual chondrosarcomatous dedifferentiation. This case broadens the histopathological spectrum of dedifferentiated adamantinoma and highlights the diagnostic challenges posed by its morphological complexity.

## 2 Case description

### 2.1 Initial presentation

A 48-year-old male initially presented with painless swelling in the right leg. The swelling worsened after exercise but subsided with rest. Plain radiography revealed a sharply demarcated osteolytic lesion in the distal cortex of the right fibula. The patient underwent tumor curettage, and a diagnosis of classic adamantinoma was made. Two years postoperatively, the patient presented to our hospital with progressive swelling and pain in the right lower limb. Physical examination showed diffuse swelling and mild tenderness in the middle and low third of the right leg, with no abnormalities in skin temperature or color. Both lower limbs were equal in length, with normal movement, muscle strength, and superficial sensation.

### 2.2 Diagnostic workup

**Figure 1:**
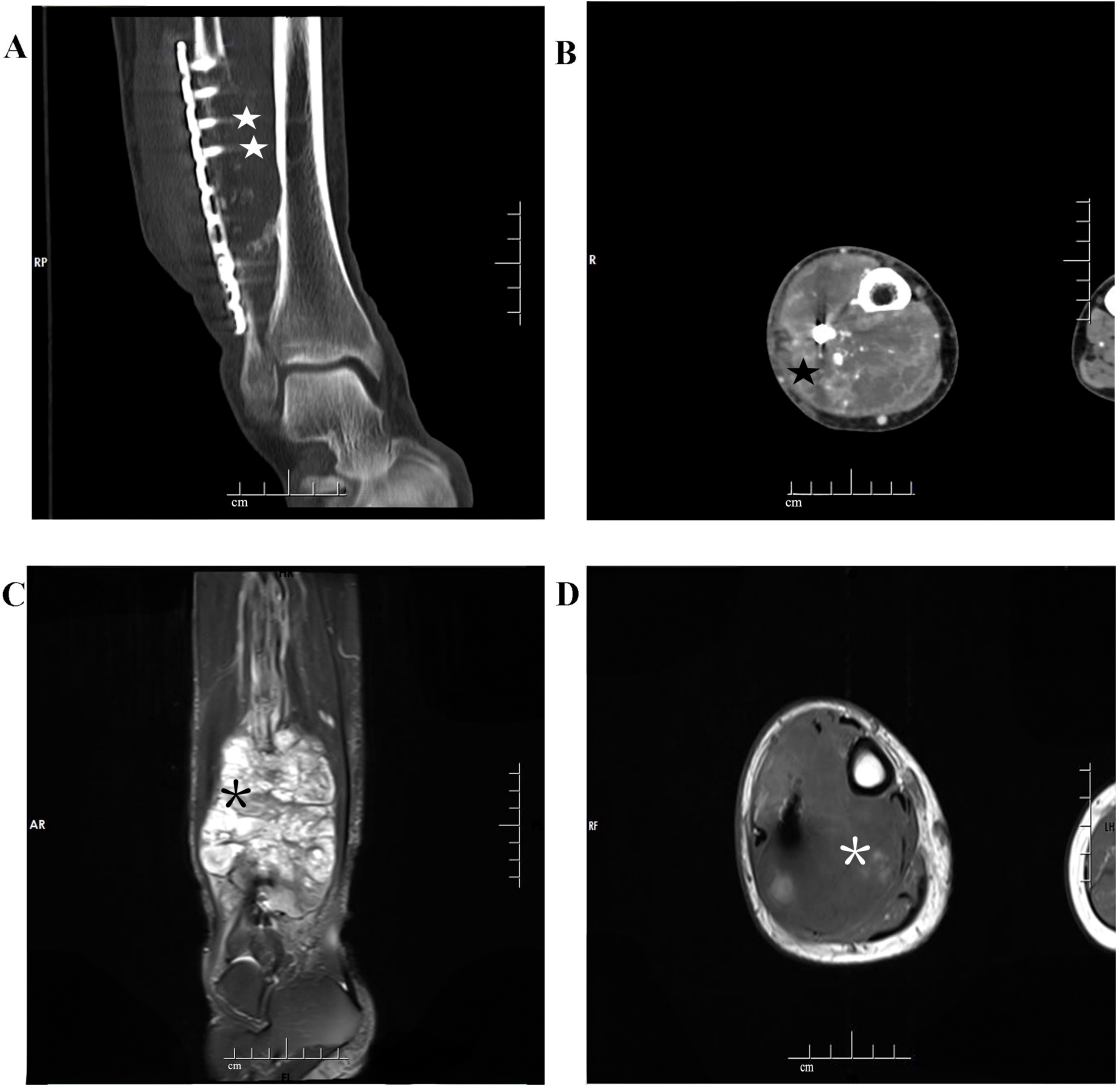
Imaging features of dedifferentiated adamantinoma in the right distal fibula. **(A)** A coronal computed tomogram (CT) scan shows a destructive lesion in the distal of the right fibula associated with the soft tissue, surrounding the previously implanted plate and screw fixation (white star symbol). **(B)** Marked enhancement is observed after intravenous administration of the Iodobitol contrast agent (black star symbol). **(C, D)** A very bright signal on T2WI (black asterisk) and low signal intensity on T1WI (white asterisk) in magnetic resonance imaging (MRI).

The computed tomography (CT) scan revealed an aggressive, solid, and destructive lesion in the distal half of the right fibula, with soft tissue involvement and calcified components encasing the previously implanted plate and screw fixation (**Figure 1A**). The mass was aggressive, eccentric, and lobulated, originating from the anterior extraosseous and encasing the mid-diaphysis of the right fibula. A marked enhancement was observed after intravenous administration of the Iodobitol contrast agent (**Figure 1B**). The tumor measured 8.0 cm × 7.0 cm in maximum axial dimension and 11.4 cm in maximum sagittal dimension. This lesion had a very bright signal on T2-weighted imaging and low signal intensity on T1-weighted imaging in magnetic resonance imaging (MRI) (**Figures 1C, D**). Plain radiograph and CT scan of the thorax were normal.

### 2.3 Treatment

**Figure 2:**
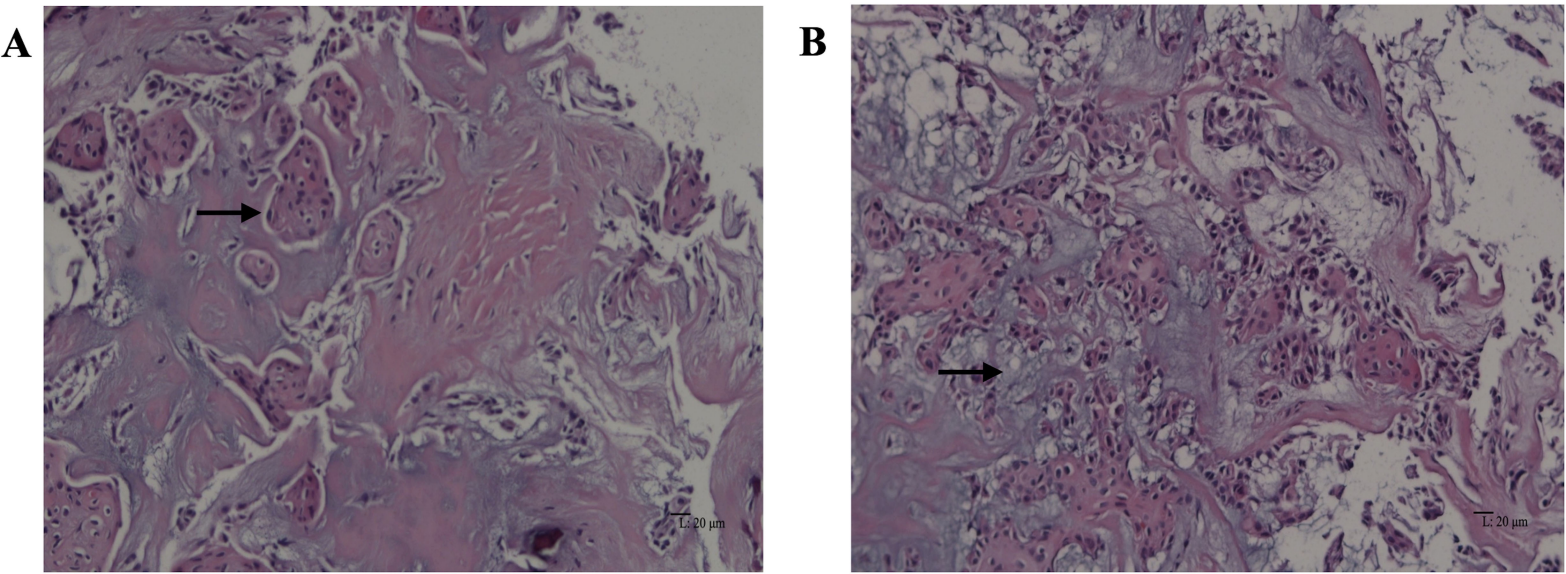
Classic adamantinoma: microscopic features of the biopsy specimen. **(A)** Squamous epithelial cells (black arrow) embedded within the fibrous stroma (hematoxylin and eosin, original magnification ×200). **(B)** Focal myxoid change (black arrow) is observed (hematoxylin and eosin, original magnification ×200).

A biopsy was performed, and the histologic diagnosis confirmed classic adamantinoma with moderate atypical epithelial cells (**Figures 2A, B**). Subsequently, a below-knee amputation was proposed.

### 2.4 Pathology findings

**Figure 3:**
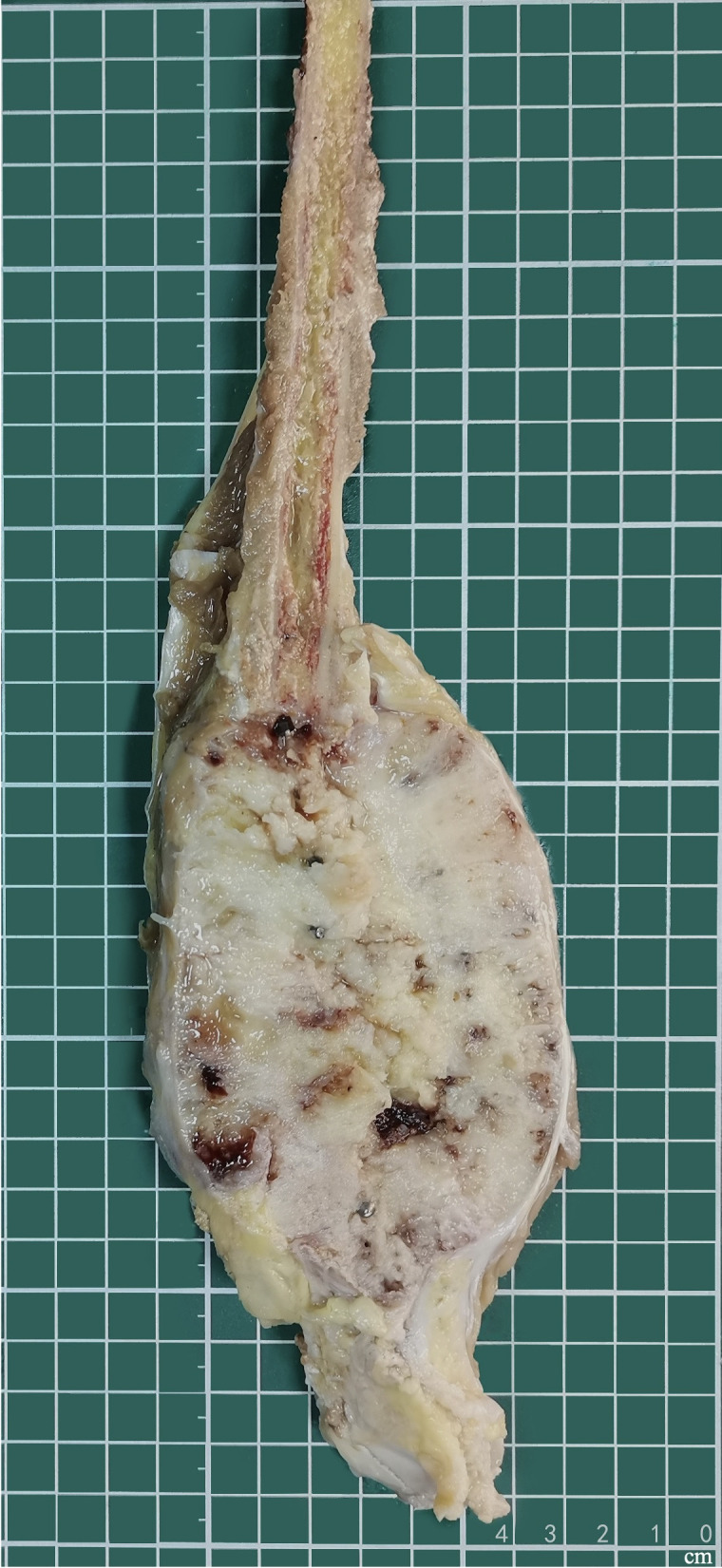
Gross feature of amputation specimen. Solid tumors involve the distal fibula, which extends into the marrow cavity and involves the surrounding soft tissue.

**Figure 4:**
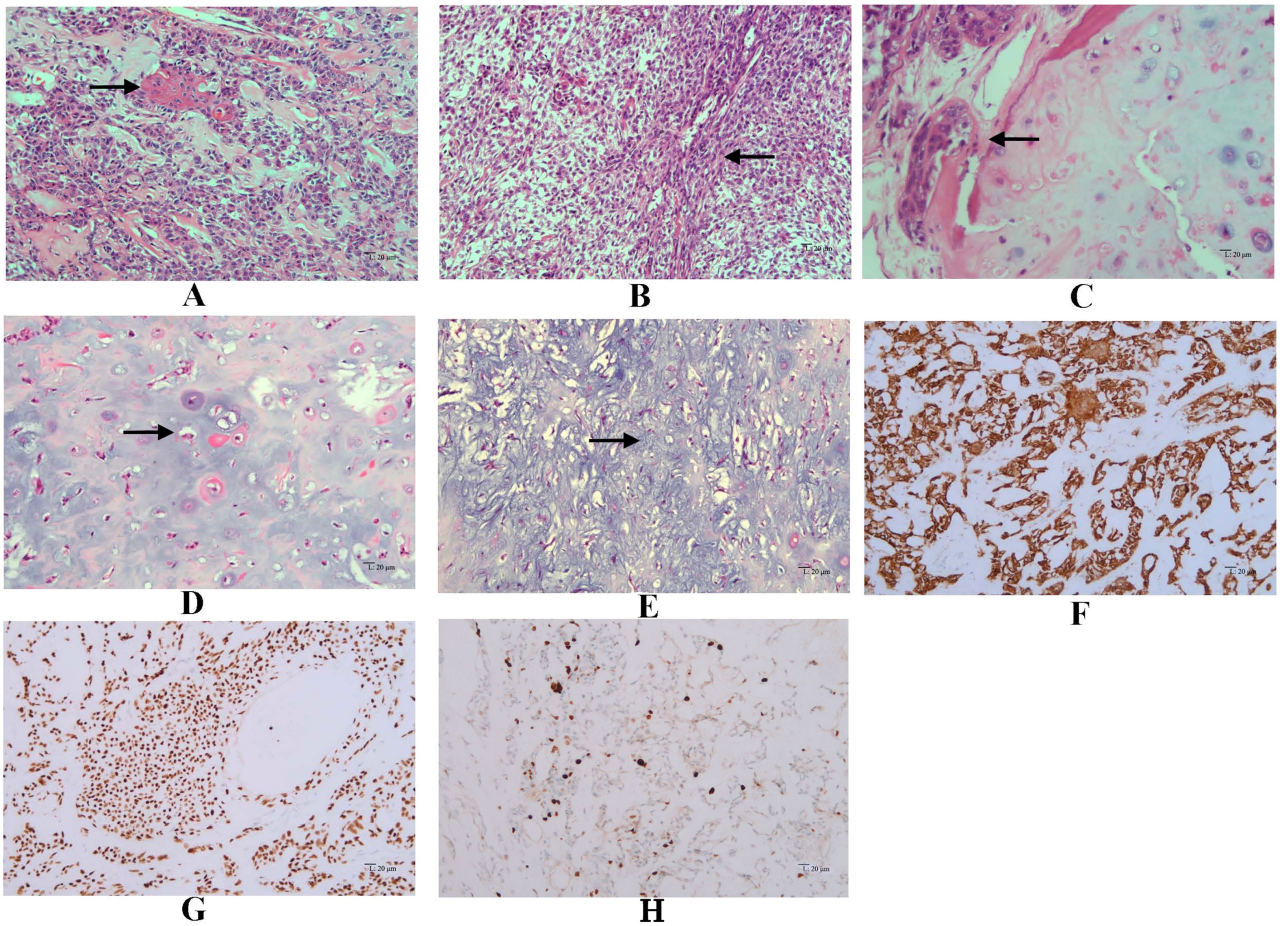
Microscopic features of dedifferentiated adamantinoma. **(A)** Features of classic adamantinoma: strings of epithelial cells embedded in the fibrous stroma (red arrow), basaloid pattern with peripheral palisading, along with squamous component (black arrow) (hematoxylin and eosin, original magnification ×200). **(B)** Spindle cells arranged in fascicles (black arrow), showing moderate nuclear irregularity, accompanied by focal myxoid change (hematoxylin and eosin, original magnification ×200). **(C)** epithelial component adjacent to chondrosarcoma component (black arrow) (hematoxylin and eosin, original magnification ×200). **(D)** Chondrosarcoma areas with moderate cellularity and abundant hyaline cartilage matrix (black arrow) (hematoxylin and eosin, original magnification ×200). **(E)** Myxoid areas (black arrow) containing malignant cartilage cells of myxoid chondrosarcoma, with nuclear irregularities in size and shape (hematoxylin and eosin, original magnification ×200). Immunohistochemistry demonstrates diffuse positivity for CK34βE12 **(F)** and P63 **(G)** in the epithelial component (EnVision, original magnification ×200). **(H)** Immunohistochemical staining of Ki-67 in tumor tissue (EnVision, original magnificatiom ×200).

Grossly, the amputated specimen revealed a destructive, solid tumor in the distal fibula measuring 12 cm × 9 cm × 7.5 cm. The tumor appeared white and fleshy, occupying the marrow cavity, destroying the cortex, and invading the surrounding periosteum and soft tissue (**Figure 3**). Areas of necrosis and bleeding were also observed. Microscopically, the lesion exhibited two distinct histologic patterns: classic adamantinoma adjacent to a dedifferentiated component. The epithelial cells displayed a basaloid pattern with peripheral palisading, and squamous and spindle cell components varied in size, displaying dispersed chromatin and infrequent mitotic figures (**Figure 4A**). Spindle cells were arranged in fascicular patterns, showing moderate nuclear irregularity, hyperchromasia, and prominent nucleoli, with focal myxoid change (**Figure 4B**). Areas with prominent hyalinized matrices were observed. The dedifferentiated component comprised a chondrosarcoma area with moderate cellularity and an abundant hyaline cartilage matrix. Myxoid areas containing malignant cartilage cells of myxoid chondrosarcoma were also observed with nuclear irregularities in size and shape (**Figures 4C-E**). The soft tissue margins and bone cut ends were free of tumors.

Immunohistochemically, epithelial cells showed diffuse positive for CK5/6, CK34βE12, CK19, and P63, while were negative for CD31, CD34, SATB2, S100, and Vimentin (**Figures 4F, G**). Immunohistochemistry staining for Ki-67 showed positive nuclear expression in approximately 10% of tumor cells, indicating a high proliferative index (**Figure 4H**). The juxtaposed sarcomatoid components were completely negative for epithelial markers while positive for S100 and Vimentin, supporting the mesenchymal differentiation (**Table 1**).

**Table 1:** Immunohistochemical profile of the tumor components.

Marker	Localization	Expression		Diagnostic Interpretation
		Epithelial Component	Dedifferentiated Component	
CK5/6	Cytoplasm	P	N	Confirms epithelial differentiation
CK34βE12	Cytoplasm	P	N	Supports epithelial differentiation
CK19	Cytoplasm	P	N	Supports epithelial differentiation
P63	Nuclear	P	N	Confirms epithelial differentiation
S100	Nuclear	N	P	Supports cartilaginous differentiation
Vimentin	Cytoplasm	N	P	Supports mesenchymal differentiation
SATB2	Nuclear	N	N	Excludes osteogenic tumors
CD31	Cell membrane	N	N	Excludes vascular tumors
CD34	Cell membrane	N	N	Excludes vascular tumors

P, positive; N, negative.

### 2.5 Follow-up

The patient remains free of recurrence or metastasis with a closed two-year follow-up.

## 3 Discussion

Dedifferentiated adamantinoma is the rarest subtype of adamantinoma, characterized by an abrupt transition from classic adamantinoma to high-grade dedifferentiated sarcomatous areas. In our case, we described a middle-aged male presenting with recurrent adamantinoma in the fibula. The initial biopsy specimen revealed only epithelial cells, which led to an underdiagnosis until further sampling identified the dedifferentiated component. Histological examination showed that the dedifferentiated component exhibited features of chondrosarcoma. Myxoid regions containing malignant cartilage cells, characteristic of myxoid chondrosarcoma, were also observed. Notably, no osteoid was identified. The dedifferentiated component showed a complete loss of epithelial features. A systematic search of PubMed, Web of Science, and Embase found no prior reports of myxoid chondrosarcomatous dedifferentiation in adamantinoma, confirming the novelty of this case.

Since the first description by Hazebag in 2003, 12 cases of dedifferentiated adamantinoma have been reported (**Table 2**) (5). Patient ages ranged from 21 to 83 years (median 40), with a slight male predominance. Clinically, patients often present with an aggressive course, including swelling, pain, pathological fracture, or a palpable mass (6). Owing to its rarity, the true incidence is likely underestimated. Unlike classic or OFD-like adamantinomas, the dedifferentiated subtype generally shows a more destructive growth pattern with cortical disruption and extension into adjacent soft tissue or the medullary cavity. Most lesions arise in the tibia (70%, 9/13), either *de novo* or as recurrences from a preexisting classic adamantinoma (5, 7). Uncommon cases have been described in the ulna and the rib. Among these patients, five cases were recurrences of a classic adamantinoma. A rare case coexisted with fibrous dysplasia (8–10). When dedifferentiated adamantinoma is identified in other skeletal sites, thorough exclusion of other primary or metastatic neoplasms is critical.

**Table 2:** Summary of reported cases of dedifferentiated adamantinoma in the literature.

Number	Author	Time	Sex/Age (year)	Location	Dedifferentiated component	Treatment	Follow-up Duration (month)	Outcome
1	Hazelbag et al. (5)	2003	F/83	Right tibia	osteosarcoma	Amputation	84	ANED
			M/63	Left tibia	Chondroblastic osteosarcoma	Excision, radiotherapy	56	Recurrence, ANED
			M/40	Left tibia	Pleomorphic cell sarcoma	Excision	14	Recurrence, metastasis, DOD
2	Izquierdo et al. (9)	2010	M/41	Left tibia	Spindle cell sarcoma	Excision, amputation, chemotherapy	12	Recurrence, metastasis, DOD
3	Nouri et al. (10)	2011	M/21	Left tibia	Spindle cell sarcoma	Amputation, chemotherapy	24	Metastasis, DOD
4	Tirabosco et al. (8)	2015	F/40	Tibia	Osteosarcoma	Unknow	7	Metastasis, DOD
			M/61	Ulna	Osteosarcoma	Unknow	15	Recurrence, metastasis, DOD
			F/32	Tibia	Osteosarcoma	Unknow	135	Recurrence, metastasis, DOD
			F/38	Rib	Osteosarcoma	Unknow	38	AWD
5	Rekhi et al. (7)	2019	M/25	Right tibia	Pleomorphic/spindle cell sarcoma	Excision	5	ANED
			F/51	Right tibia	Spindle cell sarcoma	Excision	Unknow	Unknow
6	Dong et al. (11)	2022	M/30	Left ulna	Osteosarcoma, pleomorphic cell sarcoma, mesenchymal chondrosarcoma	Excision	10	ANED
8	Present case		M/50	Right fibula	Myxoid chondrosarcoma, chondrosarcoma	Amputation	24	ANED

F, female; M, male; ANED, alive with no evidence of disease; AWD, alive with disease; DOD, died of disease.

Radiologically, dedifferentiated adamantinoma typically presents as mixed lytic and sclerotic lesions with aggressive margins, often originating within the cortex yet invading surrounding soft tissue and medullary cavity. CT is beneficial for evaluating cortical destruction and detecting metastatic disease, while MRI provides superior visualization of intramedullary and soft tissue extension.

The definitive diagnosis of dedifferentiated adamantinoma depends on histopathological examination. The epithelial cells vary significantly in size and pattern, commonly showing basaloid configurations with peripheral palisading, tubular or squamoid differentiation, and spindle cell morphology. The dedifferentiated components typically comprise high-grade sarcomas, including pleomorphic sarcoma, (chondroblastic) osteosarcoma, spindle cell sarcoma, chondrosarcoma, mesenchymal chondrosarcoma, and myxoid chondrosarcoma (11). Osteosarcomatous dedifferentiation is the most common manifestation. Immunohistochemically, epithelial cells of dedifferentiated adamantinoma express cytokeratins (CK5, CK14, CK19) and epithelial membrane antigen (EMA). The staining patterns for cytokeratins in dedifferentiated components show variability, with some cases exhibiting a reduction and others showing a complete loss. In our case, the epithelial markers were absent in the dedifferentiated area. Our observation aligns with Izquierdo’s model that complete loss of epithelial features represents a terminal stage in adamantinoma progression (9). Under this model, a tumor evolves from classic adamantinoma to a dedifferentiated adamantinoma with retained epithelial features, ultimately losing all epithelial hallmarks. This process contrasts with OFD-like adamantinoma, which is considered a more “differentiated” form. Recent studies suggest that OFD, OFD-like adamantinoma, and classic adamantinoma represent a spectrum with shared cytogenetic findings (trisomies of chromosomes 7, 8, and 12) and podoplanin expression (12, 13). Overexpression of p53 in recurrent or dedifferentiated lesions further implies a late-stage molecular driver (7, 14).

When biopsy specimens are limited, especially if fibro-osseous elements predominate, adamantinoma of long bones can be mistaken as metastatic carcinoma, fibrous dysplasia, or OFD. Conversely, basaloid or squamous foci can mimic metastatic carcinoma. A thorough clinicoradiologic evaluation, including ruling out a primary tumor elsewhere, noting the cortical location, younger patient age, and evaluating the lesion’s histopathological and immunohistochemical profile, can help exclude metastatic carcinoma. It is also crucial to obtain sufficiently large and representative biopsy specimens, ideally from the most radiolucent areas. Rarely, a false diagnosis of Ewing’s sarcoma, particularly the variant with epithelial differentiation known as adamantinoma-like Ewing’s sarcoma, may occur due to overlapping phenotypic features and immunohistochemical positive for high-molecular-weight keratin and P40. Identifying an *EWSR1/FUS::FLI1* gene fusion is pivotal in confirming Ewing’s sarcoma (15). Similarly, adamantinoma foci consisting predominantly of spindle cells may resemble intraosseous synovial sarcoma, which can be excluded by *SS18-SSX* fusion (16). Moreover, the high-grade (pleomorphic or spindle) component must be distinguished from fibrosarcoma, rhabdomyosarcomatous and leiomyosarcomatous. Hence, molecular analysis is crucial when histopathological and immunophenotypic overlap occurs. A comprehensive approach combining imaging, histology, immunostaining, and genetic tests is often necessary for a definitive diagnosis.

No standardized treatment guidelines exist for adamantinoma. Wide surgical excision (en bloc resection) with negative margins remains the cornerstone, frequently coupled with limb-salvage reconstruction. Amputation is considered for extensive recurrences or infeasible salvage scenarios but may not improve survival. Adamantinoma demonstrates poor responsiveness to chemotherapy in primary tumors, with only modest survival benefit in metastases, and shows intrinsic resistance to radiotherapy (17). Given the high invasiveness and recurrence rate of dedifferentiated adamantinoma, early and thorough surgical resection is paramount. The role of adjunctive therapies remains uncertain due to the lack of large-scale clinical evidence.

Following wide excision, about 20% of adamantinoma patients experience local recurrence, and 15%-30% develop metastases, most commonly to the lungs and/or lymph nodes (10). Risk factors for local recurrence or metastasis include positive surgical margins, male gender, younger age at onset, short symptom duration, and lack of squamous differentiation (1). However, in cases of dedifferentiated adamantinoma, the recurrence rate is 30% (4/13), and the metastasis rate is about 50% (6/13), supporting Hazelbag’s hypothrsis that sarcomatoid dedifferentiation is associated with tumor progression and aggressive metastatic behavior. Thus, prolonged follow-up is crucial.

Classic adamantinoma generally yields favorable long-term survival rates exceeding 90% at five and ten years (18). However, sarcomatous dedifferentiation usually implies tumor progression and diminished survival, reflecting a more aggressive nature of dedifferentiated adamantinoma. Among reported cases with 5 to 135 months of follow-up, approximately half of the patients (6/13) with dedifferentiated adamantinoma succumbed to disease-related events. Studies in dedifferentiated chondrosarcoma suggest that dedifferentiated areas consisting of osteosarcomatous components may have relatively better survival outcomes compared to those harboring fibrosarcomatous or undifferentiated pleomorphic sarcoma components. Similarly, in dedifferentiated liposarcoma, a higher mitotic rate correlates with poor survival (19). Larger-scale and long-term investigations are needed to clarify whether specific dedifferentiated features and/or the proportion of dedifferentiated components influence the prognosis in dedifferentiated adamantinoma.

Thus far, no definitive molecular alterations have been pinpointed in dedifferentiated adamantinoma. Nevertheless, parallels with dedifferentiated chondrosarcoma suggest that the anaplastic component shares a common precursor with the cartilaginous component but acquires additional genetic alterations. In this context, comprehensive genomic and molecular investigations of dedifferentiated adamantinoma are essential to elucidate its pathogenesis and to inform the development of targeted therapeutic strategies.

## 4 Conclusion

In summary, we report a rare case of dedifferentiated adamantinoma, characterized by an unusual chondrosarcomatous dedifferentiated component exhibiting a complete loss of epithelial histological and immunohistochemical features. Dedifferentiated adamantinoma should be suspected when an epithelial-to-mesenchymal transformation is observed in the tibia or fibula lesions. It underscores the importance of thorough sampling, close radiologic-pathologic correlation, and a multidisciplinary framework for accurate diagnosis and optimal management of complex bone tumors. Further research into the mechanisms of epithelial-to-mesenchymal transition may ultimately refine prognostication and therapy for this uncommon yet aggressive tumor.

## Data Availability

The original contributions presented in the study are included in the article/supplementary material. Further inquiries can be directed to the corresponding author.
